# Low Prevalence of SARS-CoV-2 Antibodies in Canine and Feline Serum Samples Collected during the COVID-19 Pandemic in Hong Kong and Korea

**DOI:** 10.3390/v15020582

**Published:** 2023-02-20

**Authors:** Yun Young Go, Maura Carrai, Yan Ru Choi, Christopher J. Brackman, Karina W. S. Tam, Pierra Y. T. Law, Fiona Woodhouse, Jane Gray, Ji Hun Kim, Joohyung Park, Chae Won Jeon, Hyomi Jang, Ioannis Magouras, Nicola Decaro, Samuel M.S. Cheng, Malik Peiris, Julia A. Beatty, Vanessa R. Barrs

**Affiliations:** 1 Department of Infectious Diseases and Public Health, Jockey Club College of Veterinary Medicine and Life Sciences, City University of Hong Kong, Hong Kong 999077, China; 2 Department of Veterinary Clinical Sciences, Jockey Club College of Veterinary Medicine and Life Sciences and Centre for Animal Health and Welfare, City University of Hong Kong, Hong Kong 999077, China; 3 Agriculture, Fisheries and Conservation Department, Government of the Hong Kong Special Administrative Region, Hong Kong 999077, China; 4 Society for the Prevention of Cruelty to Animals (Hong Kong), 5 Wan Shing St, Wan Chai, Hong Kong 999077, China; 5 24 h Jamsil On Animal Medical Center, Songpa-gu, Seoul 05556, Republic of Korea; 6 VIP Animal Medical Center, Seongbuk-gu, Seoul 02830, Republic of Korea; 7 Neodin Biovet Laboratory, Guri-si 11956, Republic of Korea; 8 Department of Veterinary Medicine, University of Bari Aldo Moro, 70010 Valenzano, Italy; 9 School of Public Health, LKS Faculty of Medicine, The University of Hong Kong, Hong Kong 999077, China

**Keywords:** SARS-CoV-2, COVID-19, feline, canine, seroprevalence, zoonosis

## Abstract

Severe acute respiratory syndrome coronavirus 2 (SARS-CoV-2) has affected millions of people worldwide since its emergence in 2019. Knowing the potential capacity of the virus to adapt to other species, the serological surveillance of SARS-CoV-2 infection in susceptible animals is important. Hong Kong and Seoul are two of Asia’s most densely populated urban cities, where companion animals often live in close contact with humans. Sera collected from 1040 cats and 855 dogs during the early phase of the pandemic in Hong Kong and Seoul were tested for SARS-CoV-2 antibodies using an ELISA that detects antibodies against the receptor binding domain of the viral spike protein. Positive sera were also tested for virus neutralizing antibodies using a surrogate virus neutralization (sVNT) and plaque reduction neutralization test (PRNT). Among feline sera, 4.51% and 2.54% of the samples from Korea and Hong Kong, respectively, tested ELISA positive. However, only 1.64% of the samples from Korea and 0.18% from Hong Kong tested positive by sVNT, while only 0.41% of samples from Korea tested positive by PRNT. Among canine samples, 4.94% and 6.46% from Korea and Hong Kong, respectively, tested positive by ELISA, while only 0.29% of sera from Korea were positive on sVNT and no canine sera tested positive by PRNT. These results confirm a low seroprevalence of SARS-CoV-2 exposure in companion animals in Korea and Hong Kong. The discordance between the RBD-ELISA and neutralization tests may indicate possible ELISA cross-reactivity with other coronaviruses, especially in canine sera.

## 1. Introduction

Severe acute respiratory syndrome coronavirus-2 (SARS-CoV-2), the causative agent of the ongoing coronavirus disease (COVID-19) pandemic, belongs to the genus of *Betacoronaviruses* in the family *Coronaviridae*. Coronaviruses can infect a wide range of birds and mammals, and manifest high genetic diversity due to mutations and the recombination of their genomes [1,2,3]. Several members of the family *Coronaviridae* circulate continuously in human and animal populations and mainly cause mild symptoms in humans, such as the common cold or mild enteric disease [4,5,6,7]. In contrast, the SARS-CoV and the Middle East respiratory syndrome-associated coronavirus (MERS-CoV) are highly pathogenic in humans and cause severe lower respiratory tract infections that can further develop into acute respiratory distress syndrome (ARDS) and extrapulmonary manifestations [4,8,9,10,11]. In addition, SARS-CoV and MERS-CoV, which originated in bats and infected humans via intermediate hosts, are well-known for cross-species transmission. Similarly, it is believed that the novel SARS-CoV-2 that emerged in late 2019 in Wuhan, China, has a zoonotic origin from bats, while the intermediate host has not yet been identified [12,13,14].

While the current global pandemic is driven by human-to-human transmission, concern has arisen regarding disease transmission between humans and susceptible animals as evidenced by sporadic cases of SARS-CoV-2 infection in companion, farmed, wild and captive zoological animals that have been in close contact with infected humans [15,16,17,18,19,20]. Knowing the zoonotic origin of the disease and the potential capacity of the virus to adapt to other species, it is important to understand the extent of natural SARS-CoV-2 infection of animals, in particular cats and dogs in households that are in direct contact with their owners and other household members. Natural SARS-CoV-2 infection has been sporadically detected in companion animals, with human-to-animal transmission suspected in almost all cases [16,21,22,23,24,25,26,27]. Serological surveillance of cats and dogs has been conducted in different countries since the early phases of the COVID-19 pandemic [26,27,28,29,30,31,32,33,34,35,36,37,38,39,40]. Specifically, the seroprevalence of SARS-CoV-2 in cats with an unknown history of exposure to the virus was found to be 0.015% (2/13397) in China [28], 0.69% (6/920) in Germany [29], 0.76% (1/131) in Croatia [30], 0.4% (2/500) in the Netherlands [31], 3.3% (15/451) in Italy [32], 18.9% (46/243) in Poland [33] and 7.3% (40/547) in Brazil [34]. In dogs, the seroprevalence was 0.014% (1/7159) in China [28], 0.31% (2/654) in Croatia [30], 0.2% (1/500) in the Netherlands [31], 3.3% (15/451) in Italy [32] and 16% in Poland (62/388) [33]. Not surprisingly, the seroprevalence of animals from COVID-19-affected households was generally higher than in those with unknown exposure, ranging from 10% to 12.8% in dogs and 4.5% to 43.8% in cats [25,26,27,28,29,30,31,32,33,34,35,36,37,38,39,40]. Increases in seroprevalence were also reported between the first and second waves of the pandemic; in Germany, the seroprevalence of SARS-CoV_2 increased from 0.65% to 1.36% in cats [29,39], while in the UK it increased from 0–2.2%% in cats and from 0–1.4% in dogs [40]. 

Hong Kong and Seoul are two of the most densely populated urban cities in Asia, where companion animals often live in close contact with humans. It is estimated that 241,900 (9.4%) households in Hong Kong keep dogs and/or cats [41], whereas one in three South Korean households have companion animals, with the number of pet dogs and cats reaching 8.6 million (6.02 million dogs and 2.58 million cats) [42]. In this study, we used an ELISA that detects the SARS-CoV-2 receptor binding domain (RBD) to assess the prevalence of anti-SARS-CoV-2 antibodies in 1040 cats and 855 dogs with an unknown history of exposure to the disease during the early phase of the pandemic in Hong Kong and Seoul. Positive results were confirmed using a surrogate virus neutralization (sVNT) and plaque reduction neutralization test (PRNT). In addition, we investigated the prevalence of SARS-CoV-2 RNA shedding in oronasal swabs from a subset of 301 healthy free-roaming cats presented to a trap–neuter–release desexing clinic in Hong Kong using RT-PCR.2. 

## 2. Materials and Methods

### 2.1. Sample Description

#### 2.1.1. Seoul, Korea

Residual diagnostic sera from 344 dogs and 488 cats from households of unknown COVID-19 status collected from April to July of 2020 were obtained from three veterinary practices in different districts of Seoul, Republic of Korea. All animals were sampled by veterinarians during routine healthcare visits for various reasons. For each sample, the following information was available: species, gender, age at sampling, and date of sampling. 

#### 2.1.2. Hong Kong, China

Residual diagnostic sera were collected from 511 dogs (from January 2021 until August 2021) and from 251 cats (from March 2020 to April 2021) from households of unknown COVID-19 status presenting for routine healthcare visits at a large veterinary primary care and referral hospital in Sham Shui Po, Hong Kong. In addition, serum samples, nasal and oropharyngeal swabs were collected from 301 healthy free-roaming colony cats desexed in a trap–neuter–release (TNR) program at a large animal shelter in Wan Chai, Hong Kong, from January 2021 to May 2021. 

Positive control sera were obtained from a dog and a cat admitted to the Agriculture, Fisheries, and Conservation Department (AFCD) quarantine facility because their owners were diagnosed with COVID-19. These two animals had been confirmed to be shedding SARS-CoV-2 in respiratory and fecal secretions and tested positive for serum-neutralizing antibodies using PRNT_90_ test, with titers of 1:160 for the dog and ≥1:320 for the cat [15,16]. 

For use as negative controls and ELISA optical density (OD) cut-off determination, 46 feline sera and 23 canine sera that were collected before the COVID-19 outbreaks, before January 2020, that had been stored at −80°C were used. Animal ethics approval for this study was granted by the Animal Ethics Committee of the City University of Hong Kong, approval no. A-0478. Research licenses for sampling colony cats were granted by The Government of the Hong Kong SAR, Department of Health (20–164 to 20–179).

### 2.2. Assay Description and Optimisation

All serum samples were heat-inactivated at 56 °C for 30 min before use. The sera were tested using an ELISA to detect anti-SARS-CoV-2 spike glycoprotein RBD antibodies. The protocol was based on a previously described assay with modifications [43]. Briefly, 96-well plates (Nunc MaxiSorp, Thermo Fisher Scientific, Waltham, MA, USA, cat. No. 44-2404-21) were coated with 100 ng per well of SARS-CoV-2 Spike RBD-mFc Recombinant Protein (Sino Biological, Beijing, China, Sinobiological, cat. No. 40592-V05H) diluted in PBS buffer and refrigerated overnight at 4 °C. The next day, coated plates were blocked using 100 μL of Chonblock blocking/sample dilution ELISA buffer (Chondrex Inc., Redmon, WA, USA, cat. No. 9068) for 1 h at room temperature. Heat-inactivated serum samples were diluted 1:100 in a Chonblock blocking/sample dilution ELISA buffer, with a 15 min incubation at room temperature. After 3 rounds of washing the plate with PBS containing 0.2% Tween 20, the diluted sera samples were transferred to the coated plate and incubated in a humidified chamber at 37 °C for 2 h. Washing was performed before adding horseradish peroxidase (HRP)-conjugated goat anti-feline or canine IgG antibodies (Thermo Fisher Scientific, cat. No PA1-84673 or A18763, respectively) diluted 1:2000 in PBS. The plates were incubated in a humidified chamber for 1 h at 37 °C, then washed. Subsequently, 100 μL of 3,3′,5,5′ tetramethylbenzidine (TMB) substrate (Ncm TMB One; New Cell and Molecular Biotech Co. Ltd., Suzhou, China, cat. No. M30500) was added into each well. After a 6 min (feline) or 5 min (canine) incubation, the reaction was stopped by adding 50 μL of 2 M H_2_SO_4_ solution and analyzed on an absorbance microplate reader at a 450 nm wavelength (SpectraMax ABS, Molecular Devices). Test samples were run in duplicate; each plate contained a duplicate positive control, negative control, and blank control (PBS only). For cut-off determination, the mean of the OD_450_ of negative sera collected pre-COVID-19 plus 3× and 6× the SD of the negative control readings for cats and dogs were used, respectively. 

### 2.3. Confirmatory Serological Tests: Surrogate Virus Neutralization and Plaque Reduction Neutralization Tests

Canine and feline sera that tested positive on ELISA were evaluated for SARS-CoV-2 specific-neutralizing antibodies using a commercially available sVNT assay (GeneScript Inc., Piscataway, NJ, USA) according to the manufacturer’s instructions. The OD values were read on a spectrophotometer (Molecular Devices SpectraMax ABS, San Jose, CA, USA) at 450 nm. Positive and negative controls provided by the kit were included in duplicate in every run. The quality control and validation of the results were based on the OD values for positive and negative controls falling in the recommended values. The results were interpreted as positive for SARS-CoV-2 neutralizing activity when the sVNT inhibition was calculated to be >20%, while <20% was regarded as a negative result. 

Subsequently, canine and feline serum samples with positive results in sVNT were further tested with a (PRNT in a BSL-3 laboratory at the University of Hong Kong, as previously described [15]. 

### 2.4. Detection of SARS-CoV-2 RNA Shedding in Oronasal Secretions of Healthy-Free Roaming Cats in Hong Kong by RT-PCR

To investigate the prevalence of active shedding of SARS-CoV-2 RNA in oronasal secretions collected from the 301 healthy free-roaming cats in Hong Kong, RT-PCR was performed to detect the *E* gene and *RdRp* genes as previously described [16].

### 2.5. Statistical Analyses

Data analysis was performed using GraphPad Prism software v.9.3.1 (GraphPad Software, LLC, San Diego, CA, USA). The coefficient of variation (CV) was calculated as the ratio of the standard deviation to the mean multiplied by 100. Intra-assay CV ≤ 10% and inter-assay CV ≤ 15% were considered acceptable [44]. A Pearson correlation coefficient was calculated for ELISA-positive serum samples that were further analyzed by sVNT. 

## 3. Results

### 3.1. Intra- and Inter-Assay Coefficients of Variation (CV)

The in-house SARS-CoV-2 RBD ELISA showed intra-assay CV values within acceptable limits, with CV < 10% for the canine and feline sera. The inter-assay CV of the canine ELISA was 5.66% for the positive control serum and 10.94% for the negative control serum. The inter-assay CV of the feline ELISA was 9.93% for the positive control serum and 13.28% for the negative control serum. 

### 3.2. SARS-CoV-2 Seroprevalence in Domestic Cats

**Figure 1:**
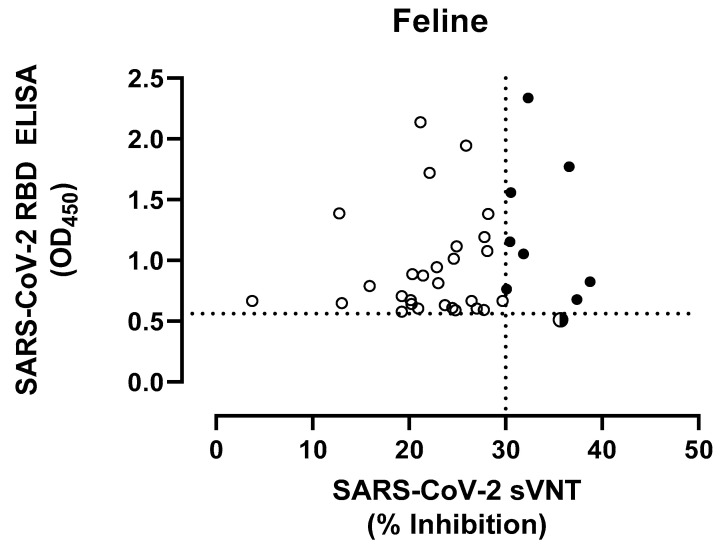
Comparison of serological results of feline samples tested by ELISA detecting antibodies to the receptor binding domain (RBD) of the SARS-CoV2 spike protein, with virus neutralization (sVNT) (see Table S1). The dotted lines show the positive cut-off levels. Cat serum samples (n = 37) are indicated in circles, whereas semi-closed circle indicates ELISA-negative and sVNT-positive (n = 1) and closed circles indicate positive samples by both assays (n = 8). Pearson correlation coefficient was calculated to determine the correlation between the reactivities of RBD ELISA vs. sVNT.

The positive control cat serum (PRNT_90_ ≥1:320) showed a mean OD_450_ value of 1.918 by RBD-ELISA. The mean OD_450_ value of 46 pre-COVID-19 feline sera was 0.196 ± 0.123, resulting in a cut-off OD_450_ of 0.564 for positive samples. Of the 488 feline serum samples from Korea, 22 samples (4.51%) were deemed to be positive for anti-S RBD antibodies by ELISA with OD_450_ values ranging from 0.606 to 2.336, while of the 552 feline sera collected in Hong Kong, 14 samples (2.54%) were positive (OD_450_ range 0.580 to 1.388). Specifically, 13 out of 251 sera (5.18%) and only 1 out of 301 sera (0.33%) sampled in veterinary primary care hospitals and animal shelters in Hong Kong, respectively, tested positive. In addition, nasal and oropharyngeal swabs collected from 301 cats of the animal shelter tested negative for the presence of SARS-CoV-2 RNA by RT-PCR. The 36 feline sera that tested ELISA-positive were evaluated using the sVNT. Nine samples, including eight from Korea (1.64%) and one from Hong Kong (0.18%), tested positive, with an inhibition ranging from 30.46 to 38.75% (Figure 1). One feline serum sample obtained from Hong Kong tested ELISA-negative (OD_450_ 0.512), but was positive by sVNT with an 35.71% inhibition. The Pearson correlation coefficient analysis confirmed the lack of correlation between RBD ELISA and sVNT.

Subsequently, the nine positive samples by sVNT were further evaluated for the presence of neutralizing antibodies by PRNT_90_ and PRNT_50_, of which two from Korea (H-C262 and H-C266) were positive. Specifically, H-C262 had a mean OD_450_ of 2.336 and 32.35% inhibition, whereas H-C266 showed a mean OD_450_ of 0.7659 and 30.10% inhibition by RBD-ELISA and sVNT, respectively. One sample (ID 155961) obtained from a veterinary primary care hospital in Hong Kong with an OD450 value of 0.5121 tested positive by sVNT with 35.71% inhibition. However, this sample was determined to be negative by PRNT_90_. Both PRNT_90_ and PRNT_90_ gave comparable results. A summary of the SARS-CoV-2-specific antibodies detected from feline sera by three serological assays is shown in Table 1.

### 3.3. SARS-CoV-2 Seroprevalence in Domestic Dogs

**Figure 2:**
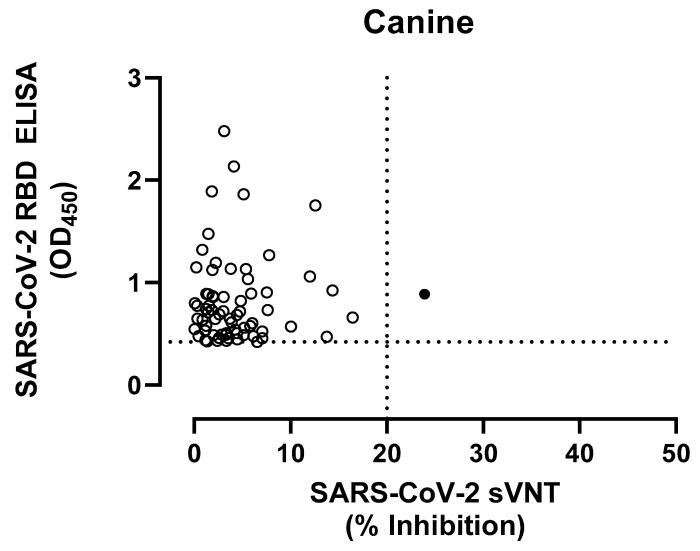
Comparison of serological results of canine samples tested by RBD ELISA and sVNT (see Table S2). The dotted lines show the positive cut-off levels. Dog serum samples (n = 36) are indicated in circles, whereas closed circles indicate positive samples by both assays.

The positive control dog serum (PRNT_90_ 1:160) showed a mean OD_450_ value of 1.762 by RBD-ELISA. The mean OD value of the negative control pre-COVID sera was 0.223 ± 0.065, yielding a cut-off of 0.610 for positive samples. Of the 344 sera from Korea, 17 (4.94%) were positive with OD values ranging from 0.632 to 2.479, while of the 511 sera collected in Hong Kong, 33 (6.46%) were positive with an OD range of 0.611 to 2.135. When the positive sera by RBD-ELISA were tested with sVNT, only one sample from Korea (H-D303; OD_450_ 0.8879) tested positive with a 23.92% inhibition (Figure 2). 

A summary of the SARS-CoV-2-specific antibodies detected from canine sera by three serological assays is shown in Table 2.

### 3.4. Prevalence of SARS-CoV-2 RNA Shedding in Oronasal Secretions of Healthy-Free Roaming Cats in Hong Kong

SARS-CoV-2 RNA was not detected in any of the samples from the 301 healthy free-roaming cats in Hong Kong, using RT-PCR to detect the E gene and RdRp genes.

## 4. Discussion

In this study, we performed a large-scale serological investigation in cats and dogs during the emergence of the COVID-19 pandemic in Seoul and Hong Kong, regions geographically adjacent to Mainland China, from where SARS-CoV-2 originated. Our results showed that the seroprevalence of SARS-CoV-2 exposure in cats and dogs in Korea was 4.51% and 4.94%, respectively, whereas SARS-CoV-2 seropositive cats and dogs in Hong Kong were 2.54% and 6.46%, respectively, as determined by RBD-ELISA. For cats, our data indicated that the seroprevalence in Hong Kong was relatively lower than in Korea, which likely reflects the difference in human incidence of SARS-CoV-2 between the two regions during the sample collection period. In Korea, pet cat samples were collected from three veterinary clinics located in different districts of Seoul from April 2020 to July 2020, where the human incidence rate was 1150 cases in Seoul during this period (total cumulative human incidence of 5,707,477 cases in the city by the end of July 2020), according to the data released by the Ministry of Health and Welfare of South Korea (available at https://ncov.kdca.go.kr/en/ accessed on 4 January 2023). The pet cat sera from Hong Kong were collected over a more extended period (March 2020 to April 2021 inclusive), encompassing the second to fourth COVID-19 waves with an average of only 28 human cases per day [45,46]. Since both Hong Kong and South Korea used aggressive public health control measures to contain COVID-19 transmission, the cumulative human infection rates were low during 2020–2021. Nevertheless, the seroprevalence among cats in both cities was comparable to other regions, including Germany (4.2%), the United Kingdom (3.3%), Italy (4.2%), and Spain (6.4%) during the first COVID-19 wave in Europe [47].

Similarly, the low seroprevalence (0.33%, 1/301) and absence of SARS-CoV-2 RNA shedding found in free-roaming colony cats in Hong Kong might be explained by the lower human incidence during the collection period, January 2021 to May 2021, which coincides with the end of the fourth wave in Hong Kong, but also by less human–cat interactions among these free-roaming cats. Interestingly, among 22 RBD-ELISA-positive feline sera from Korea, eight exhibited neutralizing activity by sVNT, while only one out of fourteen RBD-ELISA-positive sera from Hong Kong tested positive on sVNT. Serum collection from the animals in this study was carried out during 2020–21, a period where antigenic diversity of circulating strains was close to the wild-type virus used for the serological tests reported here. Thus, antigenic diversity is unlikely to have led to false negative results.

Although virus neutralization assays are considered the gold standard, little is known regarding their sensitivity compared to RBD-ELISAs in identifying SARS-CoV-2 infections in dogs and cats. The discrepant results between these two assays could be explained by the detection of different types of anti-SARS-CoV-2 antibodies by the two assays. The SARS-CoV-2 sVNT detects antibodies with neutralizing activity against the RBD of the SARS-CoV-2 spike protein [48,49]. Previously, monoclonal antibody studies demonstrated that the RBD-sVNT measures functional neutralizing antibodies, whereas the RBD-ELISA detects both binding and neutralizing antibodies [50]. In addition, it should be considered that not all neutralizing antibodies necessarily bind to the RBD, as reported by previous SARS-CoV studies that indicated antibodies raised against other regions in the S1 or S2 protein could also play a role in virus neutralization [51]. Furthermore, the difference observed in measuring neutralizing antibodies between sVNT and RBD ELISA also suggested that the level of RBD-binding IgG antibodies used alone is not reliable for evaluating the level of neutralizing capacity in companion animals. Dileepan et al. showed that the neutralizing antibodies in RBD-seropositive samples exhibited generally low titers and, in some instances, even lower than some of the RBD-seronegative samples, suggesting SARS-CoV-2 neutralizing activity in pet cats targets non-RBD regions of the S protein [52]. Nevertheless, possible cross-reaction with other feline as yet unidentified betacoronaviruses, as described in other studies [49,53], cannot be ruled out, which warrants further investigation regarding the specificity of the RBD-ELISA.

Similar to cat samples, a modified RBD-ELISA used in human epidemiologic studies was established in the laboratory to detect SARS-CoV-2 RBD antibodies in dog sera. However, the positive cut-off for dogs could not be determined using the pre-COVID-19 cohort sample values plus a three-fold standard deviation (SD), as described for the cat samples. The pre-pandemic samples exhibited low reactivity, which led to using a six-fold SD value for the positive cut-off [48]. The low reactivity in the pre-COVID-19 samples in RBD-ELISA was also described previously [54]. Despite using a high positive cut-off, a high antibody-positive rate was observed in dog sera regardless of the origin, highlighting the possible cross-reactivity with other canine coronaviruses, probably canine respiratory coronavirus (CRCoV) [45,55]. Unfortunately, the lack of negative control sera for CRCoV collected during the pandemic limited the capacity to determine the specificity of the RBD-ELISA. In this study, only one out of seventeen RBD-ELISA-positive samples from dogs in Korea was confirmed positive by sVNT, indicating a low SARS-CoV-2 infection rate in dogs, consistent with previous publications.

The data presented in this study supports previous studies showing that dogs and cats can develop neutralizing antibodies against SARS-CoV-2 [31,32,34,40,54]. However, this study has some limitations; the owners’ health status in association with COVID-19 was not known at the time of sampling of the dogs and cats presented to veterinary clinics. Additionally, except for samples collected from cats in an animal shelter in Hong Kong that were tested by RT-PCR, the status of SARS-CoV-2 shedding in pet animals was not determined. Therefore, it was not possible to determine associations between antibody levels and possible exposure to SARS-CoV-2 from infected humans, nor any perceived association of COVID-19 disease in these patients.

In summary, our data confirmed that cats and dogs in close contact with people can develop an antibody response against SARS-CoV-2 infection, and similar to other studies, the seroprevalence of SARS-CoV-2 among cats and dogs belonging to owners of unknown COVID-19 status was low [28,29,30,31,32]. This study also highlights the need to establish species-specific serological assays that can assess neutralizing antibodies in companion animals to provide accurate measurements of active infection in households and communities to ensure that all transmission opportunities are prevented.

## Figures and Tables

**Table 1:** Seropositivity of SARS-CoV-2 detected with RBD-ELISA, sVNT, and PRNT_90_ in feline sera from Hong Kong and Korea.

Species	Total Sera	Origin	Collection Date	No. of Sera	No. of ELISA Positive (%)	No. of sVNT Positive (%)	No. of PRNT Positive (%)
Cats	1040	Korea	04/2020 to 07/2020	488	22 (4.51%)	8 (1.64%)	2 (0.41%)
		Hong Kong		552	14 (2.54%)	1 (0.18%)	0 (0%)
		-Veterinary clinic	03/2020 to 04/2021	251	13	1	0
		-Animal shelter	01/2021 to 05/2021	301	1	0	0

**Table 2:** Seropositivity of SARS-CoV-2 detected with RBD-ELISA, sVNT, and PRNT_90_ in canine sera from Hong Kong and Korea.

Species	Total Sera	Origin	Collection Date	No. of Sera	No. of ELISA Positive (%)	No. of sVNT Positive (%)	No. of PRNT Positive (%)
Dogs	855	Korea	04/20–05/20	344	17 (4.94%)	1 (0.29%)	0
		Hong Kong	01/21–08/21	511	33 (6.46%)	0	0

## Data Availability

The data presented in this study are available in Supplementary Tables S1 and S2.

## Supplementary Materials

The following supporting information can be downloaded at: https://www.mdpi.com/article/10.3390/v15020582/s1, Table S1. Serological results of feline samples by sVNT and RBD-ELISA; Table S2. Serological results of canine samples by sVNT and RBD-ELISA.
